# Comparison of the playfulness of mothers of preschool children with typical development and developmental delays

**DOI:** 10.3389/fpsyg.2026.1843229

**Published:** 2026-06-09

**Authors:** Elif Açıkgöz Söğüt, Esra Betül Kölemen

**Affiliations:** Preschool Education at Faculty of Education, Sakarya University, Sakarya, Türkiye

**Keywords:** child development, developmental delay, parent–child interaction, playfulness, preschool children, typical development

## Abstract

Early childhood is a critical developmental period in which the quality of parent–child interaction shapes children’s cognitive, language, motor, and social–emotional development. Although playfulness is considered an important psychological resource in early interactions, limited research has examined how playfulness varies according to children’s developmental status and specific developmental domains. This study aimed to compare the playfulness tendencies of mothers of preschool children with typical development and developmental delays and to examine the relationship between playfulness, children’s developmental domains, and selected sociodemographic variables. The sample consisted of 152 mother–child pairs attending kindergartens and special education kindergartens in western Turkey during the 2024–2025 academic year. Data were collected using the Adult Playfulness Trait Scale and the Denver II Developmental Screening Test. The findings showed that mothers of typically developing children had significantly higher levels of playfulness than mothers of children with developmental delays. Playfulness was also significantly associated with children’s developmental domains, particularly language development. These findings highlight playfulness as an important psychosocial factor that may contribute to supportive developmental environments in early childhood.

## Introduction

The preschool period is a critical stage characterized by the highest degree of neurodevelopmental flexibility, during which environmental stimuli play a decisive role in development (Shonkoff and Phillips, 2000; World Health Organization, 2020). During this phase, children’s cognitive, motor, language, and social–emotional development are largely influenced by the family. The United Nations Convention on the Rights of the Child emphasizes the essential responsibility of parents in meeting the developmental needs of the child (Dağ et al., 2015).

Mother–child interaction is one of the fundamental determinants of early development. Attachment theory reveals that secure and responsive parenting experiences are critical for the development of self-regulation, social competence, and psychological resilience in children (Sroufe, 2005; Feldman, 2020). Bronfenbrenner’s bioecological systems model suggests that child development takes shape through processes of reciprocal interaction within the immediate environment (Bronfenbrenner and Morris, 2006). In this context, parental personality traits and psychosocial tendencies are among the key factors that determine the quality of the child’s developmental experiences (Belsky and Jaffee, 2015).

Development in the preschool period is a dynamic and multidimensional process that progresses through the reciprocal interaction of biological maturation and environmental experiences (Bronfenbrenner and Morris, 2006; Shonkoff and Phillips, 2000). Motor development in this period is a neurological and physical process involving the acquisition of gross and fine motor skills (Adolph and Hoch, 2019). Cognitive development includes executive functions and problem-solving processes (Diamond, 2013), while language development forms the foundation of social interaction (Hoff, 2006). Social–emotional development encompasses areas such as empathy, self-perception, and peer relationships (Gander and Gardiner, 2010). Each of these domains is directly impacted by the quality of parent–child interaction (Guralnick, 2017).

Screening tools used to assess children’s developmental status enable the early identification of potential delays and facilitate timely referrals. Within this scope, standardized developmental screening tests are utilized to evaluate the personal-social, fine motor, language, and gross motor domains of children aged 0–6 years (Frankenburg et al., 1992). Early identification of developmental difficulties is considered a key component of effective early intervention. Systematic developmental assessments conducted during early childhood enable professionals to detect potential developmental delays at an early stage. Early detection, in turn, supports the timely initiation of intervention processes and increases the effectiveness of developmental support provided to children (Glascoe, 2005; Shonkoff and Phillips, 2000).

Developmental delay is defined as a child’s failure to reach age-appropriate milestones in one or more areas of development (American Psychiatric Association, 2013). It has been reported that caring for children with developmental delays increases parental stress, anxiety, and caregiving burden (Hayes and Watson, 2012). The Family Stress Model proposes that parental stress may influence parenting behaviors and, indirectly, child development (Conger et al., 2002). This situation makes the indirect impact of parental psychosocial traits on child development more apparent. At this point, the concept of adult playfulness draws attention.

Playfulness is defined as an individual’s tendency to experience daily life with flexibility, creativity, and intrinsic motivation (Proyer, 2017). This trait is not limited to humor or cheerfulness; it embodies a multidimensional structure including cognitive flexibility, spontaneity, social openness, and the capacity to cope with stress. The model developed by Shen et al. (2014) explains playfulness through subdimensions such as social spontaneity, mental spontaneity, and expressive tendency. Its Turkish adaptation was conducted by Yurt et al. (2016). This underscores the importance of psychological resources based on parental personality traits.

The literature reports that highly playful individuals exhibit higher psychological well-being, life satisfaction, and stress coping skills (Barnett, 2018; Proyer, 2017). In the context of parenting, playfulness is thought to make the interaction environment offered to the child more flexible, creative, and supportive. Particularly in play-based interactions, spontaneous and creative parental participation can enhance children’s social and cognitive achievements (Zosh et al., 2018; Yogman et al., 2018). Previous studies have shown that parental playfulness may play an important role in children’s emotional and behavioral development. For example, parental playfulness has been found to function as a moderating factor that reduces behavioral problems among children with intellectual disabilities (Levavi et al., 2020). However, whether the psychological burdens experienced by parents of children with developmental delays affect their playfulness, and whether this is related to children’s developmental domains, has not been sufficiently investigated. Examining the relationship, especially between developmental screening results and mothers’ playfulness, is important for revealing the connection between parental personality traits and concrete developmental indicators. Accordingly, the aim of this study is to compare the playfulness of mothers of typically developing children ages 3–6 with that of mothers of children with developmental delays, and to examine the relationships among playfulness, children’s developmental domains, and demographic variables.

### Theoretical framework and literature review

The literature on parental playfulness and child development highlights several interconnected dimensions, including parental psychological characteristics, the quality of parent–child interactions, and children’s developmental outcomes. Previous research has examined playfulness as a personality trait associated with psychological well-being and positive social functioning. Other studies have focused on the role of playful parent–child interactions in supporting children’s cognitive, emotional, and social development. In addition, research on developmental delays emphasizes the importance of early identification and supportive family environments for children’s developmental progress. Despite this growing body of research, the relationship between parental playfulness and concrete developmental indicators among children with developmental delays has received limited attention. Therefore, examining playfulness in relation to children’s developmental domains may provide important insights into how parental characteristics interact with early developmental processes.

### Development in the preschool period

Development in the preschool period presents as a holistic structure shaped through the interactions the child establishes with caregivers. Daily routines, play experiences, and mutual regulation processes concurrently support motor exploration, language acquisition, cognitive flexibility, and social–emotional competencies (Feldman, 2020; Yogman et al., 2018). These areas constitute dynamic systems that mutually reinforce one another; thus, the quality of parent–child interaction can be reflected in all aspects of development (Blair and Raver, 2015).

Preschool education provides a planned and structured learning environment aimed at supporting these developmental domains. Quality preschool education programs offer children rich stimuli, foster cognitive and social development, and contribute to the formation of positive learning attitudes. In this process, guidance from teachers and parents plays a critical role in revealing the child’s developmental potential (Yogman et al., 2018).

Supporting development during this period affects not only learning outcomes but also school readiness levels such as self-regulation, attention control, and social adaptation (McClelland et al., 2014). In particular, experience-sensitive neurodevelopmental plasticity in the early years of life makes the impact of environmental interactions on development more pronounced. For this reason, the quality of interactions provided both in the family environment and in the context of preschool education plays a decisive role in the multidimensional progression of development. Regular assessment of developmental processes in the early period makes it possible to detect potential differences in a timely manner and to plan supportive interventions (Center on the Developing Child, 2016). Standardized developmental screening tools allow for the identification of risk indicators by examining the alignment between the child’s chronological age and developmental performance. One such tool, the Denver II Developmental Screening Test, aims to evaluate the developmental status of children aged 0–6 years in the domains of personal-social, fine motor, language, and gross motor skills in a multidimensional manner (Frankenburg et al., 1992).

### Developmental delay in the preschool period and the family

Developmental delay refers to a situation in which a child does not reach age-appropriate milestones in one or more developmental areas at the expected time (American Psychiatric Association, 2013). More often than not, this concept is considered not as a definitive clinical diagnosis on its own, but rather as a descriptive risk indicator that requires closer monitoring of the child’s development and, if necessary, referral to early intervention services (American Psychiatric Association, 2013). Because caring for a child with developmental delay or developmental disability can place additional demands on family life in terms of time, effort, and emotional regulation, increased parental stress and caregiving burden are frequently reported findings (Hayes and Watson, 2012; Woodman et al., 2015). Heightened stress can strain the parent’s psychological resources that support interaction—such as sensitivity, patience, and flexibility—and lead to changes in parenting behaviors (Feldman, 2020). Similarly, the Family Stress Model emphasizes that stress experienced by parents can indirectly affect child development through parenting processes (Conger et al., 2002; Masarik and Conger, 2017). Moreover, recent studies in the literature show that social support can mitigate the negative effects of stress and strengthen parenting self-efficacy, indicating that social support may play a significant role in the relationship between parental stress and self-efficacy (Fu et al., 2023; Yazicioğlu et al., 2024). In this context, how parents make sense of stressful life conditions and what psychological resources they draw upon to regulate daily interactions have become important topics of research. Especially personality-based traits such as cognitive flexibility, emotional openness, and the capacity for creative reframing can shape the parent’s functioning in response to stress and their style of interacting with their child. At this point, adult playfulness stands out as a potential psychological resource reflecting the parent’s tendency to approach daily life with flexibility, creativity, and intrinsic motivation (Proyer, 2017).

### Adult and parental playfulness

A review of the literature reveals that while the concept of playfulness is referred to as playfulness in children, in adults it is termed adult playfulness (Yurt et al., 2016). Playfulness is a personal disposition that arises from intrinsic motivation. It is based on a foundation closely aligned with personality. Personality is a significant factor that affects a person’s relationships with the environment and with others and shapes their behaviors.

Children raised by supportive parents are more inclined to try new things and to acquire hobbies in line with their interests and abilities. According to this widely accepted view, individuals raised by supportive parents who offer opportunities are more motivated and eager to engage in playful activities in adulthood. The support and attitudes of parents gain importance in shaping children’s initiative (Carr and ve Sequeira, 2007; Sharma, 2014). Individuals raised in an environment of secure attachment and love with their family, who can communicate healthily with their family, are described as more autonomous and unrestrained adults (Glaser et al., 2006).

In adulthood, playfulness is conceptualized as a relatively stable personality trait that reflects a person’s tendency to reframe situations with imagination, flexibility, and intrinsic motivation (Proyer, 2017). Playfulness is not limited to cheerfulness or a sense of humor alone; it encompasses cognitive flexibility, spontaneity, social openness, and the ability to transform routine situations into interesting experiences (Barnett, 2018; Proyer, 2017). Shen et al. (2014) identified the multidimensional nature of this construct by defining it through dimensions such as unrestrainedness, motivation to seek fun, and spontaneity. The Turkish adaptation of this theoretical framework has demonstrated adequate psychometric properties in terms of validity and reliability (Yurt et al., 2016). In the context of parenting, adult playfulness becomes an important characteristic that shapes the emotional climate and quality of interaction between parent and child. Parental playfulness refers to the caregiver’s level of engaging flexibly, creatively, and responsively with the child, particularly during play. Parents with high playfulness tend to spontaneously join in child-directed play, add elements of imagination, and respond appropriately to the child’s cues. These behaviors contribute to richer social interactions and a more emotionally supportive relationship environment (Yogman et al., 2018; Zosh et al., 2018). Indeed, it has been shown that responsive and flexible parenting behaviors are closely related to children’s social–emotional development (Feldman, 2020).

Studies have shown that adult playfulness is associated with higher psychological well-being, adaptive coping strategies, and relational satisfaction (Barnett, 2018; Proyer, 2017). More recent studies reveal that parental playfulness predicts positive parenting styles, higher-quality parent–child interaction, and better indicators of child adjustment (Seal et al., 2025; Shorer et al., 2025). It has been reported that parents with higher playfulness are more likely to display warmth, emotional accessibility, and behavioral flexibility; these traits closely align with responsive parenting (Belsky and Jaffee, 2015).

From a developmental perspective, parental playfulness can function as a protective factor, especially in situations of developmental risk. Flexible and spontaneous interactions can support language stimulation and joint attention processes, thereby contributing to children’s communication skills (Hoff, 2006; Yogman et al., 2018). Additionally, play-based physical interactions can foster motor development by enhancing both fine and gross motor exploration in children (Adolph and Hoch, 2019). However, in situations of increased parenting stress—which is more commonly observed in families of children with developmental delays—cognitive and emotional resources may become limited (Hayes and Watson, 2012). According to the Family Stress Model, such stress-inducing effects indirectly impact parenting behaviors, thereby also shaping child development (Conger et al., 2002). In this context, heightened stress levels may reduce the expression of playfulness in daily interactions.

Despite the growing interest in adult playfulness, there are limited studies that directly examine the relationship of parental playfulness with objective developmental domains, such as personal-social skills, fine motor skills, language, and gross motor skills. Furthermore, the role of parental playfulness in the context of developmental delays has not been sufficiently explored. Addressing this gap is important for better understanding the contribution of personality-based parenting traits to the development of preschool children.

### Sociodemographic correlates of playfulness

Findings regarding the relationship between adult/parental playfulness and sociodemographic variables generally demonstrate small and context-sensitive effects. In particular, fundamental variables such as age and gender are reported to produce only limited differences in playfulness rather than being decisive determinants; in contrast, the relationships between playfulness and indicators of well-being and adjustment are noted to be more consistent (Brauer and Proyer, 2024). Within this framework, demographic variables are often considered contextual indicators that explain how playfulness is “expressed” in daily life or under what circumstances it is supported, rather than being direct determinants of playfulness.

In the context of parenting, the importance of sociodemographic characteristics becomes more apparent when playfulness is considered alongside family processes. For example, a recent study examining parental playfulness along with role overload, parenting behaviors, and coparenting quality suggests that playfulness is not only an individual tendency but may also be a psychological resource associated with processes functioning within the family (Seal et al., 2025). Similarly, experimental/semi-experimental studies investigating the effects of parental playfulness show that playfulness can be increased even with short-term interventions and can yield outcomes related to the quality of parent–child interaction (Shorer et al., 2025). These findings support the interpretation that sociodemographic variables (e.g., working conditions, time constraints) can indirectly affect playfulness, especially through parenting load and opportunities for interaction.

Studies from the work and professional context demonstrate the relationship of playfulness with outcomes such as coping with stress and life satisfaction, particularly within the work environment; this suggests that variables such as “employment status” may influence the functional use of playfulness as a resource (Tandler et al., 2024). Additionally, new research examining the relationship of playfulness with individuals’ interests and career orientations provides evidence that the educational/occupational context may be linked to playfulness, necessitating more detailed investigation of sociodemographic patterns (Sendatzki et al., 2025).

Cultural context is also a critical variable in interpreting sociodemographic characteristics. For example, a study examining the relationship between parental playfulness and child playfulness in a different cultural sample indicates that parental playfulness may be associated with the ways parents interact with their children; therefore, sociodemographic factors (e.g., education, urban/rural, digital exposure) may play a role in ways specific to each culture (Wei et al., 2025). Finally, recent approaches framing playfulness as a “reframing lens” propose that an individual’s capacity to reframe stressful/disruptive life events may be related to playfulness, conceptually suggesting that sociodemographic conditions (e.g., economic pressure, time resources) can indirectly influence this mechanism (Shen et al., 2024).

### Research gap and research questions

Although previous studies have shown that parental playfulness is associated with positive parenting behaviors and relationship quality, it remains unclear how this trait differs among mothers of children with different developmental characteristics. While research has examined the quality of parent–child interaction in families of children with developmental delays (Chiarello et al., 2006), little is known about whether playfulness as a personality-based trait differs between mothers of typically developing children and those of children with developmental delays.

In addition, research that holistically examines the relationship between parental playfulness and multiple developmental domains—such as personal-social, fine motor, language, and gross motor skills—remains limited. Existing studies have generally focused on broad developmental outcomes, whereas the domain-specific and concurrent examination of developmental domains has received less attention (Leinz-Maturana, 2023). Considering the multidimensional nature of child development, examining the relationship between playfulness and different developmental domains separately may provide a more comprehensive understanding of how parental characteristics relate to early developmental processes. Furthermore, the association between parental playfulness and sociodemographic variables has not been consistently demonstrated in the literature. Although socioeconomic status, parental education, and family context are known to influence parental involvement and interaction quality (Haine-Schlagel and Walsh, 2015), it remains unclear whether these variables are related to parental playfulness and how they may function as contextual supports or constraints in parenting processes.

These theoretical gaps necessitate the systematic examination of playfulness among mothers of children with different developmental characteristics and across various developmental domains. Accordingly, the present study seeks to answer the following questions:

RQ1: Is there a significant difference in the level of playfulness between mothers of typically developing children and mothers of children with developmental delays?

RQ2: Is there a significant difference between playfulness tendency and children’s levels of personal-social, fine motor, language, and gross motor development?

RQ3: Does playfulness tendency differ according to sociodemographic variables?

## Method

### Research design

This study was conducted to compare the playfulness tendencies of mothers of typically developing children and children with developmental delays aged 3–6 and to examine the relationships between mothers’ playfulness tendencies, children’s developmental domains, and demographic variables. In the study, a cross-sectional correlational design, one of the quantitative research methods, was used. This design allows for the investigation of differences and relationships among variables without experimental intervention (Karasar, 2014).

Examining playfulness in the Turkish context is particularly important because parenting practices and family structures are shaped by cultural norms and social dynamics that influence parent–child interactions. In Türkiye, families play a central role in children’s early development, and parental involvement is considered a key factor supporting children’s cognitive, social, and emotional outcomes (Çaylan et al., 2021). Previous studies conducted in Türkiye have also highlighted that parental education, socioeconomic conditions, and family context significantly affect parenting practices and the quality of interaction between parents and children (Baysal et al., 2023; Bulunuz, 2022). Considering these contextual factors, examining playfulness among mothers of children with different developmental characteristics may provide culturally relevant insights into parent–child interaction processes and contribute to early developmental support practices in Türkiye.

### Study group

The study group of the research consists of children aged 36–72 months attending kindergartens and special education kindergartens located in western Turkey during the 2024–2025 academic year, as well as their mothers. Initially, 160 mother–child pairs were included in the research, but due to missing data, 8 participants were excluded from the data set, and the study was completed with 152 mother–child pairs. The sample group was formed using a purposive sampling method. Purposive sampling is a sampling method in which individuals possessing the characteristics most suitable for the purpose of the research are deliberately selected (Karasar, 2014). The process of selecting participants based on predetermined characteristics and criteria by the researcher for the study group is called criterion sampling (Büyüköztürk et al., 2013). These criteria include: the child being between 36 and 72 months old, attending preschool education, residing in western Turkey, and, according to Denver II results, displaying either typical development or having a defined developmental delay.

### Power analysis

To determine the adequacy of the sample size before the study, an *a priori* power analysis was conducted using the G*Power 3.1 program. For the independent samples t-test: Effect size (Cohen’s d) = 0.50 (medium-level effect); significance level (*α*) = 0.05; statistical power (1-*β*) = 0.80. As a result of the analysis conducted with these assumptions, the minimum required sample size was calculated as *N* = 128. The 152 participants included in the study exceeded this minimum value, indicating that the study has sufficient statistical power to detect a medium effect.

### Data collection tools

The data of the research were obtained using the “General Information Form,” the “Adult Playfulness Trait Scale,” and the “Denver II Developmental Screening Test,” all administered by the researcher.

### General information form

The form developed by the researchers includes a total of 10 questions regarding the mother’s age, level of education, employment status, spouse’s education and employment status, type of family, number of children, as well as the child’s age and gender.

### Adult Playfulness Trait Scale

Developed in 2014 by Shen, Chick, and Zinn, the scale was originally created in English to measure adults’ tendencies toward playfulness. It was adapted into Turkish and added to the literature by Yurt, Keleş, and Koğar in 2016. In international literature, the concept corresponding to “playfulness” has been translated into Turkish as “oynama eğilimi” or “eğlence eğilimi.” In this research, it is referred to as “eğlence eğilimi” (Yurt et al., 2016). The scale consists of 3 subdimensions and 19 items. The subdimensions are: “Uninhibitedness,” “Motivation for Seeking Fun,” and “Spontaneity.” While the initial self-report scale featured a 7-point Likert rating, the Turkish version uses a 7-point Likert scale (1: strongly disagree—5: strongly agree). In this research, the Cronbach’s alpha reliability coefficients obtained were:

Total scale: *α* = 0.88.

Uninhibitedness: α = 0.84.

Motivation for Seeking Fun: α = 0.81.

Spontaneity: α = 0.79.

These values indicate that the scale has sufficient internal consistency.

### Denver II Developmental Screening Test

The Denver II developmental screening test is used to monitor the development of children aged 0–6 and to enable the early identification of possible delays. It was first developed in 1967 by Frankenburg and Dodds to assist health personnel in identifying potential developmental problems in children. It has been standardized and implemented in more than 50 countries worldwide. With its widespread use, Frankenburg and Dodds revised the original screening test, resulting in the updated Denver II screening test. In Turkey, the first standardization study was conducted in 1982 by faculty members Kalbiye Yalaz and Shirley Epir from Hacettepe University’s Department of Pediatric Neurology. The Turkish standardization of Denver II was performed by Kalbiye Yalaz, Banu Anlar, and Birgül Bayoğlu. This test, designed to evaluate age-appropriate skills, is also used to assess children at developmental risk. The test consists of 134 items across 4 sections: personal-social development, fine motor skills, language development, and gross motor development. Based on the test results, children are classified as: Typical development, developmental delay.

* The Denver II Developmental Screening Test is not a diagnostic test.

### Data collection process

The study was conducted in accordance with the approval obtained from the Ethics Committee of Sakarya University (Decision No: 32/32). After ethical approval, research permits were sought to collect data in schools affiliated with the Ministry of National Education (MEB) in compliance with official regulations. Upon approval, permission was granted to conduct the research in five independent kindergartens and five special education kindergartens in western Turkey. These institutions were selected because they serve children from diverse socioeconomic backgrounds and provide both general preschool education and special education services for children with developmental delays. Including both types of institutions enabled the researchers to reach mothers of children with typical development as well as mothers of children with developmental delays within comparable educational environments. During the research process, participating mothers were provided with detailed information about the purpose, scope, and data collection process of the study; written parental consent was obtained based on voluntary participation. The mothers completed the scales used in the study as self-reports. Questions posed by participants during the implementation of the scales were answered, and necessary guidance was provided to ensure the data collection process proceeded smoothly. The Denver II Developmental Screening Test, used in the developmental assessment of children, was individually administered to each child in an appropriate physical environment by researchers who were trained and certified in the application of the test. Test administration followed standard procedures, and evaluations were made in accordance with the test manual. Participation in the study was entirely voluntary. Participants were informed that they could withdraw from the study at any time; it was guaranteed that collected data would be used solely for scientific purposes and stored in accordance with the principle of confidentiality.

### Analysis of data

The data were analyzed using SPSS 27 software. First, descriptive statistics related to sociodemographic variables were calculated, and frequency tables were created. Before analysis, the assumptions for parametric tests were examined; the normality assumption was evaluated by checking whether the skewness and kurtosis values were within the ±1.5 range, and homogeneity of variance was checked using the Levene test. To determine the differences between group means, an independent samples t-test was applied for variables with two groups, and a One-Way Analysis of Variance (ANOVA) was used for variables with three or more groups. To demonstrate not only the statistical significance but also the practical significance of the findings, effect sizes were calculated; Cohen’s d was reported for the t-test, and eta squared (η^2^) for ANOVA. In all analyses, the level of significance was set at *p* < 0.05.

### Findings

#### Mothers’ tendency toward entertainment according to children’s general developmental level

An independent samples t-test was conducted to determine whether mothers’ tendencies toward entertainment differed according to children’s general developmental level (typical development / developmental delay). The results of the analysis are presented in Table 1.

**Table 1:** Mothers’ tendency toward entertainment according to children’s general developmental level.

Dimension	Group	n	Mean	Sd	t	df	*p*	Cohen’s d
Motivation to seek playfulness	Typical development	76	39.43	3.64	7.72	150	< 0.001	1.25
	Developmental delay	76	32.88	6.45				
Uninhibitedness	Typical development	76	18.59	2.96	11.58	150	< 0.001	1.88
	Developmental delay	76	12.21	3.78				
pontaneity	Typical development	76	19.14	3.26	10.71	150	< 0.001	1.74
	Developmental delay	76	13.07	3.73				
Total playfulness	Typical development	76	77.17	7.48	13.04	150	< 0.001	2.11
	Developmental delay	76	58.16	10.27				

When Table 1 is examined, it is seen that the motivation for seeking fun, uninhibitedness, spontaneity, and total playfulness scores of mothers of typically developing children are significantly higher than those of mothers of children with developmental delays (*p* < 0.001). The effect sizes are large (d = 1.25–2.11).

#### Mothers’ playfulness according to developmental domains

The total playfulness scores of mothers were compared using an independent samples t-test, depending on whether their children had delays in personal-social, fine motor, language, and gross motor developmental domains. The findings are presented in Table 2.

**Table 2:** Mothers’ playfulness according to developmental domains.

Developmental domain	Delay status	n	Mean	Sd	t	df	*p*	Cohen’s d
Personal–social	Present	59	58.51	10.69	−8.26	150	< 0.001	1.37
	Absent	93	73.47	11.00				
Fine motor	Present	25	56.28	11.51	−5.15	150	< 0.001	1.13
	Absent	127	69.91	12.21				
Language	Present	74	57.72	10.02	−13.49	150	< 0.001	2.21
	Absent	78	77.10	7.43				
Gross motor	Present	29	54.97	11.07	−6.57	150	< 0.001	1.36
	Absent	123	70.66	11.68				

As seen in Table 2, the playfulness scores of mothers whose children do not have delays in any developmental areas are significantly higher (*p* < 0.001). The effect sizes are large. The highest effect size was observed in the language development area (d = 2.21).

#### Mothers’ playfulness according to demographic variables

In order to determine whether mothers’ total playfulness scores differ according to demographic variables, an independent samples t-test and one-way analysis of variance (ANOVA) were conducted. The analysis results are presented in Table 3.

**Table 3:** Mothers’ playfulness according to demographic variables.

Variable	Category	n	Mean	Sd	Test	Statistic	*p*	Effect size
Marital status	Single	7	68.00	17.36	t	0.07	0.945	ns
	Married	145	67.65	12.92				
Mother’s age	23–30	71	67.83	12.77	ANOVA	*F*(2,149) = 0.09	0.911	ns
	31–38	67	67.79	13.40				
	39–46	14	66.21	13.98				
Mother’s education level	Primary school	15	63.87	13.44	ANOVA	*F*(3,147) = 2.55	0.058	η^2^ = 0.05
	Middle school	21	64.90	15.31				
	High school	49	65.92	12.57				
	University	66	71.00	12.03				
Spouse’s education level	Primary school	14	62.43	8.22	ANOVA	F(3,147) = 5.48	0.001	η^2^ = 0.10
	Middle school	17	58.29	14.25				
	High school	57	71.12	12.38				
	University	63	68.38	12.99				
Mother’s employment status	Employed	46	72.70	11.45	t	3.22	0.002	d = 0.57
	Unemployed	106	65.48	13.20				
Spouse’s employment status	Employed	145	67.76	12.99	t	0.40	0.688	ns
	Unemployed	7	65.71	15.98				
Family type	Nuclear family	134	67.76	12.69	ANOVA	F(2,149) = 0.07	0.935	ns
	Extended family	11	66.27	16.09				
	Single-parent family	7	68.00	17.36				
Number of children	One child	51	69.22	13.62	ANOVA	F(2,149) = 1.91	0.152	ns
	Two children	73	68.21	13.08				
	More than two children	28	63.43	11.58				
Child’s gender	Female	75	69.01	13.77	t	1.26	0.211	ns
	Male	77	66.35	12.33				

ns, not significant.

When Table 3 is examined, it is seen that mothers’ total playfulness scores differ significantly by maternal education level, spouse’s education level, and mother’s employment status (*p* < 0.05). In contrast, there is no significant difference according to marital status, mother’s age, spouse’s employment status, family type, number of children, or child’s gender (*p* > 0.05). The effect sizes of the significant differences range from small to medium.

## Discussion

The present study examined the relationship between playfulness and children’s developmental characteristics during the preschool period. With regard to RQ1, which explored the differences in playfulness between mothers of typically developing children and mothers of children with developmental delays, the findings revealed that mothers of typically developing children had significantly higher levels of motivation for seeking fun, uninhibitedness, spontaneity, and total playfulness compared with mothers of children with developmental delays (*p* < 0.001; Table 1). The effect sizes were large (d = 1.25–2.11), indicating a substantial difference between the two groups. These results suggest that playfulness may be closely associated with the developmental context in which children grow. Mothers who display higher levels of playfulness may be more likely to engage in spontaneous, flexible, and imaginative interactions with their children, thereby creating richer opportunities for developmental experiences.

This finding is consistent with previous research emphasizing the importance of playful parent–child interactions in early childhood development. For instance, Yogman et al. (2018) highlighted that play-based interaction between parents and children contributes to multiple domains of development, including cognitive, language, and socio-emotional functioning. Similarly, Yurt et al. (2016) reported a positive relationship between mothers’ playfulness and children’s play behaviors. From a theoretical perspective, these findings can also be interpreted within the framework of Bronfenbrenner’s bioecological model and attachment theory, which emphasize the importance of responsive and emotionally engaging interactions within the child’s immediate environment (Bronfenbrenner and Morris, 2006; Feldman, 2020). Mothers with higher levels of playfulness may create more dynamic interaction environments by incorporating creativity and flexibility into daily routines and play activities.

At the same time, the findings may also be interpreted through the Family Stress Model. Previous research suggests that raising a child with developmental delays often increases parental stress and caregiving burden (Hayes and Watson, 2012; Conger et al., 2002). Increased levels of stress may reduce parents’ cognitive flexibility and limit spontaneous interaction behaviors. Therefore, the lower levels of playfulness observed among mothers of children with developmental delays may reflect contextual stress factors affecting parenting behaviors rather than solely individual personality differences.

From a practical perspective, parent support programs for mothers of children with developmental delays should directly address stress-related barriers that may limit playful and spontaneous interaction. Rather than simply encouraging mothers to play more, these programs should include low-pressure, family-centered strategies such as parent coaching, modeling responsive play, following the child’s lead, integrating playful interactions into daily routines, using symbolic play to support language development, and incorporating movement-based play to support motor development. Such approaches may help mothers experience play not as an additional caregiving burden, but as an enjoyable and manageable interaction. Additionally, peer support groups and brief psychoeducation on parental stress can strengthen social support, reduce the burden associated with parenting, and enhance parenting self-efficacy (Dunst and Trivette, 2009; Hayes and Watson, 2012; Fu et al., 2023; Yogman et al., 2018).

With regard to RQ2, which examined the relationship between playfulness and children’s developmental domains, the results demonstrated that mothers of children without delays in personal-social, fine motor, language, and gross motor domains had significantly higher playfulness scores than mothers of children who exhibited developmental delays (*p* < 0.001; Table 2). Large effect sizes were observed across all developmental areas, with the strongest difference appearing in the language development domain (d = 2.21). This finding highlights the sensitivity of language development to the quality of social interaction in early childhood. Language development is strongly influenced by communicative exchanges and verbal stimulation provided in everyday interactions (Hoff, 2006). Parents with higher playfulness may engage more frequently in reciprocal dialog, expressive communication, and verbal elaboration during play, thereby enriching children’s linguistic environments.

These findings are consistent with studies emphasizing the role of parental engagement in children’s language development. For example, Erdoğan and Temel (2023) demonstrated that mothers’ active participation and responsive communication during play activities support language development in young children. Similarly, play-based interaction provides opportunities for joint attention, emotional engagement, and communicative exchange, which are essential components of early language acquisition (Yogman et al., 2018). Although the Denver II does not provide clinical diagnoses, the domain-based screening results may offer meaningful insight into the interactional context in which maternal playfulness is expressed. The particularly strong difference observed in the language domain may be interpreted through the reciprocal nature of early communication. Language delays may reduce children’s verbal responses, symbolic play cues, and opportunities for sustained turn-taking, which may make playful interaction less predictable and more demanding for mothers. In such contexts, mothers may experience greater interactional uncertainty or stress, which can limit spontaneity, flexibility, and emotionally expressive engagement during play. This interpretation is consistent with evidence that early language development is strongly shaped by responsive social interaction and verbal stimulation (Hoff, 2006; Yogman et al., 2018), and with the Family Stress Model, which suggests that parenting stress may influence parent–child interaction quality (Conger et al., 2002; Masarik and Conger, 2017). Therefore, the lower playfulness scores observed among mothers of children with language-related delays should not be interpreted as an individual deficit, but rather as a possible reflection of the increased interactional demands associated with developmental risk.

Significant differences were also observed in the personal-social development domain. Parents who demonstrate higher levels of playfulness may create emotionally supportive environments that encourage children’s social exploration and interaction. Choi and Kim (2006) reported that mothers’ playfulness positively influences children’s social competence, while Barnett (2018) suggested that playful individuals tend to demonstrate greater emotional openness and social flexibility. These characteristics may provide children with opportunities to observe and model adaptive social behaviors.

The findings related to motor development may also be explained by the role of play in physical exploration and movement-based activities. Mothers with higher playfulness levels may be more likely to engage in activities such as outdoor play, dancing, or movement-based games that support children’s motor development. Previous research has shown that parental participation in physical activities can positively influence both fine and gross motor development in early childhood (Adolph and Hoch, 2019; Solmaz et al., 2022).

These findings have implications beyond the local context, as they are consistent with global early childhood intervention frameworks that emphasize responsive caregiving, early learning opportunities, and caregiver well-being as central components of nurturing care. The Nurturing Care Framework highlights that children’s developmental potential is supported not only through formal early intervention services but also through everyday caregiver–child interactions, including talking, playing, shared attention, and emotionally responsive engagement (World Health Organization et al., 2018). Similarly, the WHO guideline on improving early childhood development emphasizes the importance of strengthening responsive caregiving and early learning opportunities within family and community contexts (World Health Organization, 2020). In this regard, the present finding that maternal playfulness was particularly associated with children’s language and personal–social development suggests that playfulness-focused parent coaching may constitute a culturally adaptable and low-cost component of early intervention programs. Such coaching may help caregivers integrate playful, responsive, and language-rich interactions into daily routines, especially for children with developmental delays.

From a policy perspective, these findings suggest that playfulness-focused caregiver coaching could be integrated into routine developmental screening follow-up, preschool family education programs, and community-based early intervention services (World Health Organization et al., 2018; World Health Organization, 2020). After children are identified as developmentally at risk, brief parent-coaching sessions may focus on responsive talking, turn-taking, symbolic play, shared reading, singing, and movement-based play within daily routines (Yogman et al., 2018; UNICEF, 2023). Because these strategies can be delivered by trained preschool teachers, child development specialists, nurses, or family support workers, they may be feasible for both national early childhood policies and international low-resource intervention programs (World Health Organization, 2020; UNICEF, 2023). Thus, playfulness-focused coaching may represent a low-cost and culturally.

Playfulness-focused coaching may represent a low-cost and culturally adaptable strategy for international early intervention programs. Because playful interaction can be embedded into daily caregiver–child routines, such as talking, storytelling, symbolic play, and movement-based games, it does not require intensive clinical resources. This approach is consistent with global frameworks that emphasize responsive caregiving, early learning opportunities, and caregiver support as central components of nurturing care (World Health Organization et al., 2018; World Health Organization, 2020). Thus, incorporating brief playfulness-focused parent-coaching modules into preschool education, developmental screening follow-up, and community-based family support services may help reduce developmental risks, particularly in children with developmental delays.

Finally, with regard to RQ3, which examined whether playfulness varies according to sociodemographic characteristics, the findings indicated that playfulness differed significantly according to spouse’s education level and mothers’ employment status, whereas no significant differences were found in relation to marital status, mothers’ age, spouse’s employment status, family type, number of children, or the child’s gender (Table 3). The finding that employed mothers had higher playfulness scores may be explained by broader social participation, role diversity, and psychosocial resources associated with work life. Employment may provide mothers with opportunities for adult interaction and access to support networks outside the family, which may buffer parenting stress and enhance parenting efficacy (Fu et al., 2023; Yazicioğlu et al., 2024). In addition, expansionist perspectives on multiple roles suggest that participation in different roles may provide individuals with additional sources of competence, self-esteem, and emotional resources rather than simply increasing role strain (Barnett and Hyde, 2001; Greenhaus and Powell, 2006). Accordingly, employed mothers may experience greater role diversity and temporary psychological distance from intensive caregiving demands, which may reduce parenting-related burnout or role overload. This may support greater flexibility, spontaneity, and motivation for playful engagement with children. The absence of significant differences across several demographic variables supports previous research suggesting that playfulness is a relatively stable personality trait that may not strongly depend on demographic characteristics (Proyer, 2017; Pinchover, 2017). Similarly, Brauer and Proyer (2024) reported that demographic factors generally explain only a small proportion of variation in adult playfulness.

However, the significant differences observed according to spouse’s education level may reflect broader family interaction patterns. Families with higher educational backgrounds may provide more cognitively stimulating environments and encourage interactive parenting practices. Previous studies have shown that parents with higher educational levels tend to participate more actively in children’s play and provide richer interaction experiences (Craig, 2006; Lindsey and Mize, 2001). Similarly, the finding that working mothers demonstrated higher playfulness levels may be associated with broader social participation, role diversity, and increased opportunities for psychological well-being. Overall, the findings suggest that playfulness is not merely an individual personality trait but also a relational and contextual factor that shapes the interaction environment in which children’s developmental experiences occur. By influencing the quality of parent–child interaction, playfulness may contribute to children’s language, social, and motor development during the preschool period.

## Conclusion

This study reveals that parental playfulness is significantly and systematically related to the development of preschool children. The relationships between the concrete developmental domains measured by the Denver II and playfulness indicate that parental interaction style can shape the quality of a child’s language, social, and motor experiences. The findings suggest that playfulness is a psychosocial variable largely independent of demographic characteristics of the child but related to the developmental context. Although the cross-sectional design does not allow for causal interpretations, the results show that parental playfulness is a trait open to intervention and support. Therefore, including components that strengthen play-based and spontaneous interaction in parent support programs for the preschool period may contribute to reducing developmental risks.

### Recommendations

The findings of this research show that mothers of children with developmental delays tend to have lower playfulness. It is important that developmental monitoring processes are regularly conducted during the preschool period, that early intervention is provided in the event of possible delays, and that families are guided in practices to support development in the home environment. In particular, it is recommended to increase role and symbolic play to support language development and to include games involving physical movement to support motor development.

It is recommended that future studies focusing on parental playfulness be conducted with broader and diverse socio-cultural samples. Including fathers, incorporating children’s temperament and behavioral characteristics into the model, and developing interventions to enhance parental playfulness would contribute to the literature. Moreover, it is suggested to investigate the relationship between digital game preferences and parental playfulness, as well as to conduct studies with different developmental diagnosis groups.

### Limitations

This study has several limitations that should be considered when interpreting the findings. First, the research was conducted only in preschools and special education preschools located in western Turkey during the 2024–2025 academic year; therefore, the results may not be fully generalizable to different geographical regions or educational contexts. Second, the study sample consisted solely of children aged 36–72 months and their mothers, which limits the ability to examine the role of other family members, such as fathers or other caregivers, in parent–child interactions. In addition, the study relied on the sub-dimensions measured by the Denver II Developmental Screening Test and the Adult Playfulness Trait Scale, and therefore the findings are limited to the constructs assessed by these instruments. Finally, based on the results of the Denver II Developmental Screening Test, children were classified only as typically developing or as having developmental delays; thus, the findings do not reflect differences among specific developmental diagnoses or varying levels of developmental difficulty.

## Data Availability

The raw data supporting the conclusions of this article will be made available by the authors, without undue reservation.
